# Risk factors for wound infection in health care facilities in Buea, Cameroon: aerobic bacterial pathogens and antibiogram of isolates

**DOI:** 10.11604/pamj.2014.18.6.2304

**Published:** 2014-05-02

**Authors:** Akoachere Jane-Francis Tatah Kihla, Palle John Ngunde, Mbianda Soupsop Evelyn, Nkwelang Gerard, Roland Ndip Ndip

**Affiliations:** 1 Department of Microbiology and Parasitology, Faculty of Science, University of Buea, Buea, Cameroon; 2 Laboratory for Emerging Infectious Diseases Faculty of Science, University of Buea, Buea, Cameroon; 3 Department of clinical science, Faculty of health Sciences, University of Buea, Buea, Cameroon; 4 Department of Biochemistry and Microbiology, Faculty of Science and Agriculture, University of Fort Hare, Alice, 5700, South Africa

**Keywords:** Wound infection, antibiotic susceptibility, co-morbidity, bacterial pathogens, Cameroon

## Abstract

**Introduction:**

Wound infection is a significant clinical challenge in hospitals in developing countries where proper healthcare delivery is hampered by limited resources. This study investigated the antibiotic susceptibility pattern of bacteria causing wound infection and risk factors for infection among hospitalized patients in Buea, Cameroon, to generate findings which could drive reformation of policies on infection control.

**Methods:**

Aerobic bacteria were isolated from 212 swabs collected from patients with clinically diagnosed infected wounds. Risk factors for wound infection were investigated. Antibiotic susceptibility of isolates was determined by disk diffusion technique. The Chi-square test was employed to determine significant differences in isolation and distribution of organisms in various specimens. Differences were considered significant at P < 0.05.

**Results:**

Twelve bacteria species were isolated from 169 (79.7%) specimens. Staphylococcus aureus, Pseudomonas aeruginosa and Klebsiella pneumoniae, the predominant isolates in all wound types exhibited a high preponderance of multidrug resistant strains. High rate of infection was attributed to lack of constant water supply and breakdown of sterilization equipment during the study period. Highest diversity of pathogens occurred in open wounds. There were no significant differences (P>0.05) in isolation of pathogens with respect to age, gender and wound type. Co-existing morbidity increased risk of wound infection. Isolates were susceptible to fluoroquinolones and resistant to oxacillin.

**Conclusion:**

Wound infection with resistant bacteria constitutes a significant cause of morbidity in the study area. Findings reiterate the need to strengthen infection control and drug dispensing policies, and greater collaboration between microbiologists and medical practioners to stem the spread of resistant bacteria.

## Introduction

Despite the progress made with respect to infection control and wound management, wound infection still remains a serious and significant clinical challenge particularly in developing countries where wound site infections are a major source of post operative illness, a cause of death among burn patients [1] and accounts for approximately a quarter of all nosocomial infections [2]. These infections have resulted in increased trauma in patient, prolonged hospitalization, increased hospital cost as general wound management practices become more resource demanding.

The progression of a wound to an infected state involves a multitude of factors including old age, repeated trauma, blood perfusion, immune suppression and coexisting morbidity all of which impair wound healing, increasing the risk of infection. Other risk factors reported include gender, surgical technique, lifestyle, hospitalisation which increases risk of infection by drug resistant organisms, and length of operation [3]. The type, site, size, and depth of the wound and the combined level of virulence expressed by the types of microorganisms involved have also been reported to facilitate wound infection [4]. Therefore, knowledge of risk factors associated with infections could help strengthen efforts to reduce their occurrence, thus reducing morbidity and mortality for these infections.

The control and management of infection is a complex and important aspect of wound care. Although antibiotics have been of great value in treatment and in prophylaxis to prevent infections, the timing of administration, choice of antimicrobial agent, durations of administration have clearly defined the value of antibiotics in reducing wound infections [2]. Even though treatment especially in life-threatening situations is usually empiric employing broad-spectrum antibiotics, increasing rates of antibiotic resistance among pathogens have been a major impediment to the success of empiric treatment, resulting in treatment failure hence increasing treatment cost. In addition, the spectrum of pathogens circulating in any health care setting has been shown to change with time and even between various departments [5]. Thus routine surveillance for pathogens and their susceptibility to antibiotics is of paramount importance not only to reinforce strategies for successful wound infection control and management but in the control of antibiotic usage to stem the emergence and spread of resistant bacteria. Comprehensive data on aetiology of infected wounds in Buea and their antibiotic susceptibility patterns as well as risk factors for infection are lacking. Ndip et al. [6–8] and Nkwelang et al. [9] characterized specific pathogens recovered from wound swabs and other clinical specimens in healthcare settings in Buea. Here, we report findings from a study conducted to determine the risk factors for infection, the distribution and antibiotic susceptibility patterns of bacterial pathogens in various wound types. The study is important to generate findings that would guide the strengthening of policies on infection control, empiric antibiotic treatment and control of antibiotic use.

## Methods

### Study setting, design and data collection

This study was carried out in Buea, a cosmopolitan town situated on the eastern slopes of Mount Cameroon. The climate in Buea tends to be humid, with the neighbourhoods at higher elevations enjoying cooler temperatures while the lower parts experience a hotter climate. Although Buea has a population of about 200,000 inhabitants, there is an expected rapid annual increase in population size since Buea hosts the first Anglo-Saxon University in Cameroon and many other institutions of higher learning. Provision of health care in Buea as in other parts of the country is a joint effort of the public and the private sector, the bulk burden is borne by the public sector which is non-profit making and subsidizing health care cost. This has attracted a good patient turn out, making public hospitals the major health care providers. The private sector comprises individuals and religious organizations. Two health care facilities participated in this study: Mount Mary Health Center and the Regional Hospital Anex. Mount Mary Health Centre is a private health care facility with a Surgical Department of 46 bed capacity. The Regional Hospital Annex from where the majority of study participants were recruited is government-owned and has a Surgical Department with a capacity 50 beds. All patients with septic wounds during the period October 2010 to March 2011 were sampled. Wound swabs from different anatomical sites of hospitalized patients clinically diagnosed with wound infection were collected just before wound dressing. Microbiological characteristics of swab specimens from burns, ulcer, open wounds and post operative wounds were analyzed and their susceptibility to commonly prescribed antibiotics tested. Data were recorded on age, sex, and wound type. Information on co-existing morbidity including diabetes and HIV status of patients was obtained from patients ‘ records. Only patients who gave consent to participate in the study were enrolled. Ethical approval of the study was obtained from the management boards of participating hospitals as well as from the Regional Delegation of Public Health for the South West Region.

### Sample collection

Swabs were collected from wounds during dressing before the wound was cleaned using an antiseptic solution. A total of 212 specimen comprising swabs from burns (3), ulcer (15), open wound (165) and postoperative wounds (29) were obtained from different anatomical sites of hospitalized patients. Samples were collected only from patients clinically diagnosed with wound infection. Clinical diagnosis was based on the presence of purulent discharge or signs of inflammation (erythema, pain, tenderness, warmth or induration). The specimen was collected using sterile Calcium alginate cotton swabs avoiding contamination with skin flora. Only one sample was collected from each patient. Samples were transported to the laboratory in Aimes transport medium following standard methods [10].

### Isolation and Identification of bacterial pathogens

Specimens were inoculated onto Blood agar, MacConkey agar, Nutrient agar and Mannitol Salt agar and plates incubated aerobically at 37°C for 24 to 48 hours. Pure cultures were presumptively identified based on their morphology, colonial and Gram staining characteristics [11]. Their identity was confirmed by the oxidase, catalase, coagulase, motility, growth on Kliger iron agar and the Analytical Profile Index (API) 20E assay (Biomerieux SA, Marcy E ‘Etiole, France).

### Antibiotic susceptibility testing

The disc diffusion (Kirby Bauer) technique which conforms to the recommended standard of the Clinical Laboratory Standards Institute [12] was employed as previously described. Briefly, a small inoculum of each pure bacterial isolate was emulsified in 3mL sterile normal saline in Bijou bottles. The turbidity of cell suspension was adjusted to correspond to 0.5 Mc Farland standard containing 1.8 x 108 cfu/mL. Twenty microlitres (20 µL) of the inoculum was dispensed on the surface of Mueller-Hinton (MH) agar (Scharlau Chemie S.A, Spain) plate and ramified with a sterile spreader and the plates were allowed to dry for 3-5 minutes. Antibiotic discs with the following drug concentrations: gentamicin (10 µg), erythromycin (10 µg), ceftriazone (30 µg), ceftazidime (30 µg), ampicillin (10 µg), augmentin (30 µg), oxacillin (1 µg), co-trimoxazole (25 µg), ofloxacin (30 µg), norfloxacin (10 µg), pefloxacin (5 gµ), doxycyclin (30 µg), chloramphenicol (30 µg) and aztreonam (30 µg) ( Becton, Dickson and Company, Sparks, USA) were applied on the surface of the plates at least 15mm apart from the edges of the plates to prevent overlapping of inhibition zones. The plates were incubated at 37 °C for 24 hours and the diameter of zones of inhibition were measured and compared with diameters of the control organism E. coli ATCC 25922 to determine susceptibility or resistance [12].

### Data analysis

Statistical Package for Social Science (SPSS) version 10.0 (2008) used to analyze data. The Chi-square (χ^2^) test was employed to determine significant differences in isolation and distribution of organisms in various specimens. Differences were considered significant at P < 0.05.

## Results

### Patients ‘ profiles

A total of 212 patients presenting with various types of wounds were enrolled in the study from October 2009 to March 2010. Their ages ranged from 7 months to 80 years with 89 (42%) being females and 123 (58%) males. Majority of the pateints presented with open wounds (165/212=77.8%) while those with burns (3/212=1.4%) constituted the least. Flame burn was the cause of burn among these patients. Twenty patients had co-morbidity, either HIV infection (14/212= 6.6%) or diabetes (6/212=2.8%) (Table 1).

**Table 1:** Risk factors of wound infection

Variable	Category	No Analyzed	No. Positive (%)
Age (years)	0-15	25	21 (84)
	16-30	92	75 (81.5)
	31-45	54	43 (79.6)
	46-60	22	14 (63.6)
	>60	19	16 (84.2)
Gender	Female	89	73 (82)
	Male	123	96 (78)
Co-existing Morbidity	HIV	14	12 (85.7)
	Diabetes	6	6 (100)
Wound Type	Burns	3	3 (100)
	Ulcers	15	13 (86.7)
	Post-operative	29	23 (79.3)
	Open Wounds	165	130 (78.8)

### Prevalence of bacteria in population

**Figure 1:**
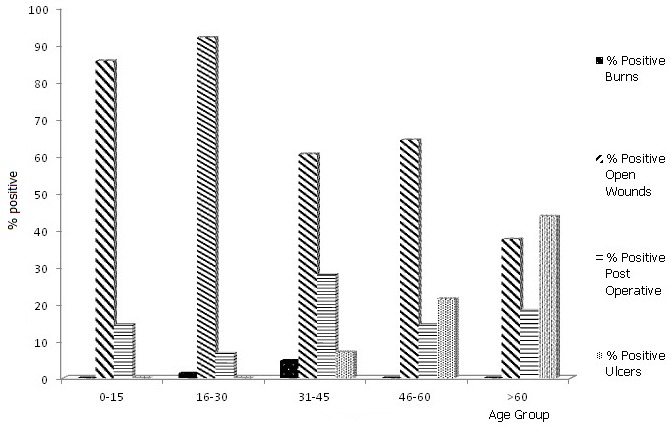
Distribution of bacteria in different wound types with respect to age group

Bacteria were isolated from 169 (79.7%) specimens (Table 1). Forty- three (20.3%) specimens had no bacterial growth. Overall, the difference in isolation between wound types was not significant (χ^2^=1.302; df=3; p=0.729). The highest rate of isolation was from the age group>60 (84.2%) followed by 0-15 years (84.0%) and least (63.6%) in individuals 46-60 years (Figure 1). However, the difference was not significant (x^2^=4.225; df=4; p=0.376). Foot ulcer constituted a major source of isolation in the age group >60, while open wound was the main source of isolation in the other age groups (Fig. 1). There was a significant difference in isolation from different sample sources within each age group (χ^2^=183.920; df= 12; p=0.0001). With respect to gender, bacteria were isolated more from females (82%, 73/89) compared to males (78%, 96/123). The difference however, was not significant (χ^2^=0.504; df=1; p=0.478). The prevalence of bacteria in samples was also analyzed in relation to coexisting morbidity such as diabetes and HIV infection. Of the 14 samples from HIV infected patients, 12 (85.7%) were infected. All cases of diabetes had wound infection (100%, 6/6). Amongst the different types of specimens analyzed, burns had the highest isolation rate of 100% (3/3) while open wounds had the least (78.8%) Amongst the different types of specimens analyzed, burns had the highest isolation rate of 100% (3/3) while open wounds had the least (78.8%).

Gram-negative organisms were the major causes of infection. Twelve species of bacteria were isolated from specimens (Table 2). Staphylococcus aureus (24.8%) was the most common isolate followed by Pseudomonas aeruginosa (23.1%) while the least isolated was Serratia sakazakii (0.6%). S. aureus and P. aeruginosa were isolated from all sample sources (Table 3). Enterobacter aerogenes, Hafnia alvei, Serratia sakazakii and Streptococcus sp. were isolated exclusively from open wounds. Majority of isolates were obtained from open wounds (76.9%, 130/169). The only isolates from burns were S. aureus and P. aeruginosa. All samples had monobacterial infection. There was no significant difference in the distribution of bacteria in the various types of wounds (χ^2^ =19.299; df =33; p=0.972).

**Table 2:** Distribution of bacterial isolates in various wound types

Isolate	Wound type Frequency of Isolation (%)				Total (%)
	Burns	Ulcers	Post Operative	Open wounds	
*S. aureus*	1 (2.1)	3 (6.3)	7 (14.6)	37 (77.0)	48 (28.4)
*P.aeruginosa*	2 (5.1)	4 (10.3)	3 (7.7)	30 (76.9)	39 (23.1)
*K. pneumonia*	0 (0)	2 (9.1)	4 (18.2)	16 (72.7)	22 (13.0)
*Enterobacter cloacae*	0 (0)	1 (4.7)	1 (4.7)	19 (90.5)	21 (12.4)
*Escherichia coli*	0 (0)	2 (20)	2 (20)	6 (60)	10 (5.9)
*Serratia marcescens*	0 (0)	0 (0)	2 (20)	8 (80)	10 (5.9)
*P. mirabilis*	0 (0)	1 (12.5)	3 (37.5)	4 (50)	8 (4.7)
*E. aerogenes*	0 (0)	0 (0)	0 (0)	3 (100)	3 (1.8)
*S. rubideae*	0 (0)	0 (0)	1 (33.3)	2 (66.7)	3 (1.8)
*H. alvei*	0 (0)	0 (0)	0 (0)	2 (100)	2 (1.2)
*Streptococcus. sp*.	0 (0)	0 (0)	0 (0)	2 (100)	2 (1.2)
*S. sakazakii*	0 (0)	0 (0)	0 (0)	1 (100)	1 (0.6)
**Total%**	3 (1.8)	13 (7.7)	23 (13.6)	130 (76.9)	169 (100)

(χ^2^ =19.299; df =33; p=0.972)

**Table 3:** Antibiotic resistance patterns exhibited by isolates

Isolates	Drugs resistance patterns	Number of isolates exhibiting pattern (%)	Number of isolates per pattern (%)
*E. cloacae*	SXT^R^ AMP^R^	14 (15.2)	20 (21.7)
*P. mirabilis*	SXT^R^ ATM^R^	4 (4.3)	
*S. rubidaea*	SXT^R^ ATM^R^	2 (2.2)	
*S. sakazakii*	SXT^R^ NOR^R^	1 (1.1)	1(1.1)
*E. aerogenes*	SXT^R^ AMP^R^ ATM^R^	1 (1.1)	1(1.1)
*S. marcescens*	DXT^R^ NOR^R^ C^R^	3 (3.3)	3 (3.3)
*Streptococcus sp*.	GEN^R^ SXT^R^ NOR^R^ AMP^R^	1 (1.1)	1 (1.1)
*K. pneumoniae*	SXT^R^ DXT^R^ C^R^ AMP^R^ ATM^R^	16 (14.4)	37(40.2)
*P. aeruginosa*	SXT^R^ DXT^R^ C^R^ AMP^R^ ATM^R^	21 (22.8)	
*S. aureus*	CAZ^R^ SXT^R^ DXT^R^ C^R^ E^R^ AMP^R^	29 (31.5)	29 (31.5)
**Total**		92	92

### Antibiotic susceptibility

Susceptibility of isolates to drugs ranged from 0 to 100% depending on the organism and drug (Table 4). All isolates (100%) were susceptible to ofloxacin and pefloxacin but resistant to oxacillin. Other active drugs were ceftriazone (94.3%), gentamicin (97.2%), ceftazidime (89.7%), norfloxacin (76.7%) and augmentin (76.4%). Low susceptibility was recorded for chloramphenicol (6.5%), erythromycin (7.1%), co-trimoxazole (22.6%), aztreonam (27.4%), doxycycline (34.2%) and ampicillin (39.6%).

**Table 4:** Antibiotic susceptibility of isolates (%)

*Isolate*	OOFX	CAZ	GEN Nn	SXT	PEF	CRO	DXT	NOR	C	E	AUG	AMP	OX	ATM
*E. coli*	100	100	100	60	100	100	100	100	100	10	100	80	0	100
*E. cloa*	100	100	100	9.5	100	100	90	100	4.8	4.8	100	10	0	52.4
*E. aerog*	100	100	100	33.3	100	100	66.7	100	33.3	33.3	100	66.7	0	66.7
*P. mirab*	100	100	100	25	100	50	87.5	100	12.5	12.5	100	75	0	0
*K.pneum*	100	90.9	100	0	100	100	13.6	90.9	4.5	4.5	72.7	4.5	0	0
*H. alvei*	100	100	100	50	100	100	100	100	000	0	100	50	0	0
*P.aerug*	100	87.2	74.4	0	100	100	15.4	100	2.6	7.7	76.9	7.7	0	0
*S.marces*	100	100	100	60	100	100	10	30	10	10	90	60	0	60
*S. rubide*	100	100	100	33.3	100	100	66.7	100	00	0	66.7	66.7	0	0
*S. sakazi*	100	100	100	0	100	00	100	0	0	0	0	0	0	0
*Staph.aur au*	100	47.9	87.5	0	100	81.3	20.8	50	4.20	2.1	60.4	4.2	0	0
*Strept.sp*	100	50	50	0	100	100	100	50	05	0	50	50	0	50
**Total%**	100	89.7	92.7	22.6	100	94.3	34.2	76.7	6.5	7.1	76.4	39.6	0	27.4

**Abbreviation**: **OFX**, ofloxacin; **CAZ**, ceftazidime; **GEN**, gentamicin; **SXT**, co-trimoxazole; **PEF**, pefloxacin; **CRO**, ceftriaone; **DXT**, doxycyline; **NOR**, norfloxacin; **C**, chloramphenicol; **E**, erythromycin; **AUG**, augmentin; **AMP**, ampicillin; **OX**, oxacillin; **ATM**, aztreonam

Ninety-two (54.4%) isolates exhibited resistance to at least two drugs (multidrug resistance) (Table 4). Eight patterns of multi-drug resistance (MDR) emerged based on resistance to two or more antibiotics excluding oxacillin. Sixty-six (71.7%) isolates were resistant to five or more drugs including oxacillin. The predominant resistance pattern SXTR DXTR CR AMPR ATMR was observed in Klebsiella pneumoniae and P. aeruginosa which was exhibited by 40.2% (37/92) of these pathogens. Multi-drug resistance was commonly encountered in S. aureus, P. aeruginosa and K. pneumoniae, which constituted the principal pathogens.

## Discussion

Regardless of the advances in technology and wound management techniques in modern times, wound infection is still a major concern among health care practitioners particularly in the developing world where health care cost remains high and human and financial resources to run health care facilities are grossly inadequate [13]. This has necessitated the frequent surveillance of pathogens causing infections, their susceptibility to antibiotics empirically prescribed for eradication of infection and an appraisal of risk factors of wound infection so as to update strategies for infection control and guide therapy.

Of the 212 specimens analyzed, bacteria were isolated from 169 (79.7%). The high rate of isolation indicates that wound infection could be a serious problem in health care settings in our study area and calls for timely measures of infection control. Water supply in participating hospitals during the time of study was not regular. In the main hospital where most of the study participants were recruited, there was not water at water supply had been interrupted for some time and there was no water in the hospital. Water was fetched from any source (non-conventional) for use in the wards and theatres. This affected general hygiene in the hospitals; more especially even pre-operative scrubbing in the theatres and cleaning of theatre equipment and materials was not done with clean water. These factors could have contributed to the high risk of wound infection reported in our study. Forty-three (43) specimens (20.3%) had no bacterial growth. This could be due to normal healing process where the bacteria might have been over-whelmed by the body ‘s defense mechanism. It is also possible that some organisms could have been anaerobic and as such were missed as cultures were incubated aerobically. This condition could therefore not support the growth of such organisms. We analyzed some contributing factors to wound infection such as wound type, age, gender and co-morbidity (diabetes and HIV infection). Identification of risk factors will facilitate infection control and reduce morbidity from these infections. The rate of infection with respect to wound type was as follows: burn wounds>ulcers>post operative wounds>open wounds (Table 1). Our results corroborate the findings of Ekrami & Kalantar [14] who reported burn wound infection as the most common infection in hospitals. The susceptibility of burn wound to opportunistic infections has been associated with several factors including the presence of coagulated proteins, the absence of blood-borne immune factors, and the vascularity of the burn wound [1]. There was no significant difference in contamination of various wound types (P≥0.05) (Table 1). Burn wounds do not seem a major concern in our study area as we sampled only three burn patients throughout our study. It is noteworthy that up to 79.3% of surgical wounds were infected. This high rate of post-operative infection could be explained by some factors that prevailed in study sites at the time of study. In one of the hospitals, patient shaving in elective cases was done about 24 hours or more prior to the procedure, rather than immediately before surgery. This long latent time between shaving and surgery has been identified [15] to increase post-operative infection. In addition, the sterilization machine in the hospital which served as the main source of study participants broke down at one point during the study. Non-conventional sterilization methods were used during this period. This compounded the effect of lack of water in the hospital and thus increased the risk of post-operative infection.

We observed the highest rate of infection of 84.2% in individuals >60years old although there was no significant difference (P≥0.05) with age (Table 1). Our findings agree with the results of Kaye et al. [16] who identified increasing age in adults as a risk factor for surgical site infection. We attribute our findings to immunologic senescence associated with increased age. Foot ulcers were the main wound type in the age>60 (Fig. 1). On the contrary, the high occurrence in the age groups 0-15 (84.0%) could be due to poor hygiene.

More females (82%) had infected wounds than males (78%) although the difference was not significant (P>0.05) (Table 1). On the contrary, Sorensen et al. [3] have implicated male gender as a risk factor for wound and tissue complication and associated their finding to a lesser collagen production and reduced wound healing capacity in men. We investigated more males (123) than females (89). Differences in sample size could have resulted in a lower prevalence in females. Underlying diseases noted in some of our study participants were HIV infection and diabetes. Specimens from 12 of the 14 HIV infected patients (85.7%) were infected with different bacterial pathogens. This underscores the effect of HIV infection in weakening the immune system of patients making them susceptible to infection. All specimens from diabetic patients were infected as well. (Table 1). Recent reports [17] reveal diabetic wounds to be significantly more susceptible to infection compared to none diabetic wounds. Cellular changes associated with diabetes including impaired leukocyte function, inadequate migration of neutrophils and macrophages to the wound due to compromised tissue perfusion predispose diabetic individuals to increased risk of wound infection. All diabetic patients sampled (6/6, 100%) had foot ulcers. Studies in some parts of Cameroon [18] have reported a high mortality rate in diabetic patients due to foot ulcer infection. Foot ulceration and infection are among the most common and serious complications of diabetes mellitus with an associated high mortality rate and the leading risk factors for amputation [19]. Thus prevention of infection, prompt diagnosis and treatment are paramount in the prevention of morbidity, especially amputation.

Although Gram-negative aerobic and facultative bacilli constituted the majority of our isolates, S. aureus (28.4%) was the leading cause of wound infection in our study area. This was followed by Pseudomonas aeruginosa (23.1%) while S. sakazakii (0.6%) was the least (Table 2). Studies elsewhere [1, 5, 20] have reported these organisms as the most prevalent in infected wounds. Other studies have isolated anaerobic bacteria [19] and fungi [1] in addition to aerobic bacteria from wounds. The high prevalence of infected wounds together with the diversity of bacteria causing these infections gives credence to the value of regular surveillance for nosocomial pathogens in our study area. Analyzing the distribution of isolates in the various wound types, it was interesting to note that S. aureus and P. aeruginosa were the only isolates recovered from all wound types and only pathogens isolated from burns (Table 2). We have previously isolated multi-drug resistant strains of these pathogens form various clinical and environmental specimens in the same study area [7, 9] indicating that they could pose clinical challenges in health care settings in our study area. S. aureus and P. aeruginosa have been recognized for their ability to produce potentially destructive virulence factors. Open wounds constituted the main source of pathogens as isolates were present with high frequencies compared to other wound types. We are of the opinion that this could be due to the fact that the bacterial flora of open wounds is seldom static, usually changing with the appearance of new organisms and disappearance of old ones. Enterobacter aerogenes, Hafnia alvei, Streptococcus. sp. and S. sakazakii were isolated solely from open wounds. All wounds had monobacterial infection. Previous studies in neighbouring Nigeria [21] have reported a high prevalence of monomicrobial than polymicrobial infections in wounds yet others have reported a high prevalence of infections with polymicrobial aetiology [22] with some of these involving both aerobic and anaerobic organisms [23]. Microbial synergy may increase the net pathogenic effect and hence the severity of infections. Anaerobic bacteria and fungi could have been present in our samples but were missed out as we did not analyze for them. This constituted a limitation to this study and could explain our detection of infections solely with monomicrobial aetiology.

Ofloxacin and pefloxacin were the most active antimicrobial agents as all isolates were susceptible. Our findings agree with recent reports from the study area [7, 9] identifying these agents as the most active and therefore the best drugs for treatment or for prophylaxis. Their high cost restricts indiscriminate use hence their high potency against pathogens. Other active drugs were ceftriaxone (94.3%), gentamicin (92.7%) ceftazidime (89.7%), norfloxacin (76.7%) and augmentin (76.4%) (Additional file 1). Thus to preserve the efficacy of the fluoroquinolones for future use, we recommend the use of these agents. We did not determine the minimum inhibitory concentrations of potent antibiotics. This constituted another limitation to our study. The most inactive drugs were oxacillin (0%), chloramphenicol (6.5%), erythromycin (7.1%), co-trimoxazole (22.6%) and aztreonam (27.4%). Previous studies in study area have reported P. aeruginosa [7] and S. aureus [9] resistance to some of these agents. Our data suggest that these drugs are of very limited value in the prophylaxis or empiric treatment of wound infections. Thus, a regular update of antibiotic susceptibility through continuous surveillance is essential to maintain good infection control programmes, thereby improving the overall infection-related morbidity and mortality.

Analysis of antibiotic resistance patterns of isolates gave eight patterns of resistance among isolates (Table 3) with a high preponderance of multi-drug resistant bacteria in the study area. Majority of isolates (66/92=71.7%) exhibited resistance to at least five drugs. Of utmost concern is the fact that our most predominant isolates, S. aureus (CAZR SXTR DXTR CR ER AMPR), K. pneumoniae (SXTR DXTR CR AMPR ATMR) and P. aeruginosa (SXTR DXTR CR AMPR ATMR), exhibited resistance to six and five drugs respectively (Table 3). Hence treatment of wound infection in our study area could be a challenge. Resistance of these pathogens to antibiotics has been high even in developed countries as such were among the pathogens listed by IDSA [24] as urgently requiring new and effective therapies as they cause severe morbidity and mortality. The high rate of multi-drug resistant isolates is probably due to the widespread empiric use of these broad-spectrum antibiotics in study area for the treatment of other bacterial infections. These drugs are relatively cheap and are sold by unauthorized persons and without prescriptions. Their high level of misuse accounts for the high levels resistance observed. The risk that wound patients carrying antibiotic resistant organisms pose to others is unknown. However, dressing changes alone have been shown to disperse significant number of bacteria to the air [25]. Since wound patients have high contact with health care staff, they may thus serve as potential reservoirs for cross contamination [9]. In study area, due to lack of qualified personnel, majority of staff in surgical department are nursing assistants, some of whom are volunteers. Because these individuals are not competent they provide poor quality care. This, in addition to their non-complicance to aseptic practices, increasing the risk of wound infection. Thus, recruitment of qualified nursing staff as well as an effective infection control policy is urgently required is hospitals in study sites. These will reduce or eliminate pathogenic and/or antibiotic resistant organisms and prevent the establishment of these drug-resistant organisms as the predominant flora in the hospital setting.

## Conclusion

On the basis of our findings, wound infection by multidrug resistant bacteria is a great concern in healthcare settings in the study area and reiterates the need for a strong collaboration between medical practitioners and microbiologists to improve treatment outcomes; regular update of infection control strategies; the use of antimicrobial agents or drug combinations with wider spectra of activity for prophylaxis or treatment of wound infection, constant supply of potable water to ensure high standards of hygiene and reduce infection. The use of natural products or non-antimicrobial methods for treatment is highly recommended as resistance to these is non-existent or negligible.
